# From Evidence to Action: Addressing Micronutrient Gaps among Women of Reproductive Age in Malawi Using the Comprehensive Nutrient Gap Assessment Framework

**DOI:** 10.1016/j.cdnut.2026.109436

**Published:** 2026-07-11

**Authors:** Zione Kalumikiza-Chikumbu, Bridget Mkama-Maliya, Gareth Osman, Alexander A Kalimbira

**Affiliations:** Department of Human Nutrition and Health, Bunda College, Lilongwe University of Agriculture and Natural Resources, Lilongwe, Malawi

**Keywords:** Malawi, women of reproductive age, micronutrient deficiencies, nutrient gap, food sources

## Abstract

**Background:**

Despite limited and irregular micronutrient data collection in resource-poor countries like Malawi, existing evidence remains underutilized despite its potential to guide policies and programs addressing persistently high micronutrient deficiencies.

**Objectives:**

This study applied the Comprehensive Nutrient Gap Assessment (CONGA) methodology to identify priority micronutrient gaps among Malawian women of reproductive age (WRA) and to determine locally available foods capable of substantially contributing to closing these gaps.

**Methods:**

The assessment followed CONGA procedures and included the following: *1*) a systematic review of existing micronutrient data; *2*) weighted scoring of available evidence using criteria of recency, representativeness, relevance, and sample size; *3*) quantitative and qualitative evaluation of nutrient burden; and *4*) classification of each nutrient as having a clear (consistent evidence) or potential (inconclusive evidence) gap. To identify food-based solutions, household consumption data from the 2019‒2020 Malawi Fifth Integrated Household Survey (*n* = 9296 WRA households) were analyzed using the adult female equivalent metric to determine locally available foods with the potential to provide ≥50% of WRA micronutrient requirements per 100 g edible portion.

**Results:**

Clear micronutrient gaps were identified for calcium, iron, selenium, and zinc, whereas potential gaps were found for vitamin B1, vitamin B12, vitamin C, and phosphorus. Small fish, gathered wild greens, and dairy emerged as nutrient-dense foods capable of addressing several of these gaps; however, their current consumption remains low. Cereals and vegetables constituted the most consumed foods among WRA households, yet they were insufficient to meet requirements.

**Conclusions:**

Substantial micronutrient gaps persist among Malawian WRA, emphasizing the need for targeted interventions to improve access to and intake of nutrient-rich foods. Priority actions include strengthening nutrition education and dietary diversification, increasing the availability and affordability of nutrient-dense foods, and expanding fortification and biofortification initiatives. Further research is required to clarify the burden associated with micronutrients classified as having potential gaps.

## Introduction

In resource-constrained countries like Malawi, data on micronutrient status are limited and collected irregularly, largely because of the high cost and logistical complexity of conducting biochemical surveys. To date, 4 national micronutrient surveys have been conducted in Malawi in 2001, 2009, 2015–2016 [1], and 2024, with a report for the 2024 survey yet to be released. The surveys have largely focused on the prevalence of 3 micronutrient deficiencies (MNDs)—vitamin A, iron (Fe), and iodine (I)—owing to their historical public health significance [2]. This is despite recognition that the burden of MNDs spans beyond the 3 nutrients and includes calcium (Ca), magnesium (mg), selenium (Se), thiamine (vitamin B1), riboflavin (vitamin B2), niacin (vitamin B3), pyridoxine (vitamin B6), folate (vitamin B9), cobalamin (vitamin B12), ascorbic acid (vitamin C), and tocopherol (vitamin E) [3]. The 2015–2016 Malawi National Micronutrient Survey included assessment of the status of zinc (Zn), vitamin B12, and vitamin B9 [1], a recognition of the expanding need to consider other MNDs beyond the traditional three. Additionally, in 2019, the status of Se deficiency among women of reproductive age (WRA) was published [4], further underscoring the need to expand monitoring beyond the traditional micronutrients.

Vitamin A deficiency has long been known as a problem of public health significance since the 1980s, following a study that revealed a 22% prevalence among 650 children [5]. Its prevalence in Malawi’s first National Micronutrient Survey conducted in 2001 was notably high at 59%, but subsequently declined to 22% in 2009 and further to 4% in 2015‒2016 [6], with the probability of near elimination [7]. From being a deficient country, Malawi has improved its population’s I status following the 1995 salt iodization legislation, which mandated iodization of all salt for human and animal consumption. As the proportion of households with iodized salt has increased, urinary I concentrations (UIC) have also been increasing. The 2015‒2016 Malawi Demographic and Health Survey reported median UIC of 268 *μ*g/L and 271 *μ*g/L for school-aged children and nonpregnant WRA, respectively, suggesting adequate I nutrition. Regarding Fe deficiency, there has been a steady decline from 61% in 2001 to 22% in 2015‒2016 among children under 5 y [6]. In contrast, Zn deficiency remains widespread, ranging from 60% in children aged <5 y to 69% in adolescent girls aged 10 to 14 y based on biomarker data. Among WRA, 41% depicted vitamin B12 depletion, whereas vitamin B9 deficiency was estimated at 8% [6].

Collectively, Malawi’s national micronutrient surveys have demonstrated a persistent prevalence of selected MNDs alongside notable declines in specific deficiencies such as vitamin A and I. This is attributable to targeted public health interventions, for instance, the mandatory large-scale fortification of foods including sugar and cooking oil, and supplementation campaigns [8]. However, evidence from these national surveys is limited in scope, focuses on a few micronutrients (vitamin A, Fe, and I), and leaves out other micronutrients, such as Ca, Se, and vitamin B1, vitamin B2, and vitamin B3, whose deficiencies are likely to be high [4,[9], [10], [11]]. Furthermore, although acknowledging that micronutrient status data generation is costly and thus irregularly conducted, the available data are underutilized to inform priorities of focus. This perpetuates a cycle of data generation and publication with limited uptake for improving the micronutrient burden. This underutilization may also discourage further investment in data collection, as it can create the perception that such data are not essential for decision making, even though limited analytical capacity and weak data-use systems may be underlying contributors. For resource-constrained countries such as Malawi, making effective use of available data is therefore critical. Our study sought to strengthen the micronutrient data value chain in ways that promote the uptake of existing evidence to inform actions for improved micronutrient status of Malawian WRA. This analysis was undertaken on the premise that inadequate dietary intake is a major underlying cause of MNDs in resource-constrained settings [9]. Addressing MNDs among WRA is especially critical given their profound implications on maternal, fetal, and infant health, economic development, and overall well-being [12].

We applied the Comprehensive Nutrient Gap Assessment (CONGA) methodology, a structured and novel approach developed by Beal et al. [13], to identify the public health significance of nutrient gaps among Malawian WRA. In this study, the term “nutrient gap” refers to a shortfall in the consumption or availability of essential nutrients that may increase risk of inadequate nutrient status, deficiency, and adverse health outcomes [13]. The nutrient gap approach, other than biomarker (deficiency data) alone, promotes more effective synthesis, interpretation, and use of available evidence to inform actions aimed at improving the micronutrient status in a population group. Additionally, we identified evidence gaps where research is needed to comprehensively understand the micronutrient burden in this population group. To complement these analyses, we used food consumption data derived from Malawi’s Fifth Integrated Household Survey (IHS5) to identify the best food sources to address existing nutrient gaps. Our assessment focused on 17 micronutrients whose intakes are critical for WRA, namely Fe, Zn, Ca, I, Se, Mg, phosphorus (P), copper (Cu), and vitamin A, vitamin B1, vitamin B2, vitamin B3, vitamin B6, vitamin B9, vitamin B12, vitamin C, and vitamin E [14]. The analysis supports the Government of Malawi’s sustained commitment to combat MNDs as evidenced by the 2003 national plan of action for micronutrient malnutrition control [15] and the subsequent 2018‒2022 national multi-sector micronutrient strategy [16].

## Methods

We employed the CONGA methodology to systematically assess micronutrient gaps among Malawian WRA. The CONGA approach involves searching the literature for evidence, weighting each data point based on a predefined criterion, assigning both quantitative and qualitative ratings to the public health burden of each nutrient, rating the certainty of evidence for each nutrient, and then validating it qualitatively [13]. The step-by-step process used to generate the study outputs is detailed in the subsequent sections, and the complete data, including the CONGA evidence scoring, score adjustments, and final scores, are available in Supplemental Data 1 (Excel workbook).

### Steps in the application of the CONGA methodology

#### Step 1: literature search and data extraction

We conducted a systematic literature search to identify relevant data on micronutrient status and dietary adequacy, focusing on the following 5 evidence types: *1*) biological, clinical, and functional markers; *2*) nutrient adequacy of individual diets; *3*) nutrient adequacy of household diets; *4*) nutrient adequacy of national food supplies; and *5*) nutrient-informative food-group intake at the individual or household level. Additional evidence sources that did not fit into these 5 categories, such as food groups rich in micronutrients or data on micronutrient supplementation coverage, were also considered when deemed relevant. Evidence was drawn from multiple national and international sources, including the Malawi micronutrient survey, published local studies, and global databases such as Demographic and Health Survey (DHS) STATcompiler (dhsprogram.com), the global dietary database, Food and Agriculture Organization Corporate Statistical Database (FAOSTAT), and the global nutrition report. Searches were conducted using Google, Google Scholar, and PubMed, with key terms including “nutrient inadequacies,” “nutrient intakes,” “dietary intake,” “food group intakes,” “micronutrient deficiencies,” “dietary quality,” “dietary patterns,” “women of reproductive age,” “nutrient gaps,” and “Malawi.” The search was restricted to English-language publications and grey literature, including both national and subnational sources. Retrieved records were sequentially screened through review of titles, abstracts, and full texts to identify studies assessing nutrient inadequacies among WRA or broader female populations, based on the predefined CONGA evidence types. Inclusion criteria were as follows: data collected over the past 20 y (i.e., from 2005 onward), sample size ≥50, and ≥10% representation of the target geographic population. Studies focusing on highly specific or nonrepresentative populations (e.g., hospitalized or otherwise vulnerable clinical groups) were excluded. Both national and subnational studies were included; however, subnational data points were assigned lower weights during analysis to account for their limited representativeness. From 14 data sources identified through this process, we extracted a total of 67 data points, 58 of which aligned with the 5 primary CONGA evidence types and were included in the quantitative burden assessment. These data points formed the basis for subsequent weighting, rating, and triangulation procedures described below.

The evidence base was drawn from multiple sources with variation in geographic coverage and population characteristics. Representativeness was accounted for through the CONGA weighting system, with nationally representative data and data closely aligned with the target population assigned greater weight. Most data points were nationally representative or modeled at national or subnational levels, whereas a small proportion were derived from subnational studies (∼20%), including 1 study covering 10 of the 28 districts in Malawi. All included data were collected within the past decade and were based primarily on cross-sectional surveys, including nationally repeated surveys where available. Although the primary focus of this study was nonpregnant, non-lactating WRA, not all data sources were disaggregated by physiological status. In such cases, broader population-level, household-level, or food supply data were included to complement available evidence. Nevertheless, data points that most closely matched the age, sex, and physiological characteristics of the target population were prioritized through higher weighting, as described in subsequent sections.

#### Step 2: implied nutrient burden rating

We reviewed all data points extracted from the various studies and assigned an Implied Nutrient Burden (INB) score (categorized as negligible, low, moderate, or high) based on suggested prevalence and mean ranges for commonly available population-level indicators from all 5 evidence types. Each indicator was mapped to a burden category using predefined cutoffs derived from the CONGA guideline ranges (Supplemental Table 1). For instance, a high INB rating was assigned to a data point with a folate deficiency prevalence of ≥20% (defined as serum folate concentrations <10 nmol/L).

#### Step 3: assignment of metadata weights and calculation of weight scores

We assigned metadata weight scores based on a defined criterion. Data points with metadata that were most recent, representative (national vs. subnational), had larger sample sizes, and relevant for WRA were considered most valuable or robust and ultimately assigned higher weight scores. We excluded all data points from other evidence types, those whose data collection was done >10 y ago (i.e., before 2015), had a sample size of <50, and were for a different demographic group other than WRA. To generate overall weight scores for each data point, the evidence type metadata weight scores were multiplied by the combined weight scores of the other 4 metadata categories (i.e., recency, representativeness, sample size, and demographic relevance).

#### Step 4: calculation of the quantitative nutrient gap burden score

The quantitative nutrient gap burden score for each nutrient was calculated by combining the implied nutrient gap burden (step 2) with the metadata weight scores (step 3), using only data points from the 5 primary CONGA evidence types. To generate the quantitative nutrient gap burden score for each nutrient, the implied nutrient gap score was multiplied by its corresponding metadata weight, and the summation was divided by the sum of the weights.

#### Step 5: assignment of qualitative nutrient gap burden ratings

We reviewed the quantitative nutrient gap burden scores together with data from other evidence types and from supplementary sources to qualitatively assign burden ratings. Nutrients were classified as having negligible (<0.5), low (0.5‒1.49), moderate (1.50‒2.49), or high (≥2.50) burden based on their scores. This step involved decision making by 4 experts to either maintain or deviate from the quantitatively derived rating. Any deviations were documented and justified to ensure transparency.

#### Step 6: certainty of evidence rating

We evaluated the certainty of evidence for each nutrient gap using a 3-tiered classification (Supplemental Table 2): low certainty (limited, low-quality, or conflicting evidence), moderate certainty (reasonably consistent and representative evidence), and high certainty (strong, consistent, and well-supported evidence). This classification was informed by step 3, which suggests that data points with metadata that are most recent, representative, relevant, and have a larger sample size are the most robust and weighed more heavily. It also assessed the level of disagreement between different data points to ascertain the severity of micronutrient gaps among WRA and applied qualitative expert judgment that considered all evidence to determine data reliability. Any deviations were documented and justified to ensure transparency in the final certainty ratings.

#### Step 7: qualitative validation of ratings with experts and local stakeholders

We gathered a group of technical experts from the micronutrients and nutrition-sensitive agriculture technical working groups under the National Nutrition Coordinating Committee to review the findings generated from this process. The purpose of this engagement was to validate the findings, resolve discrepancies, and build consensus on the final nutrient gap burden and certainty ratings. Any disagreements were discussed, reviewed, and documented, and final ratings were recorded after reaching consensus. Following this expert review, adjustments to the quantitative burden and certainty ratings were made where necessary based on consensus, with all changes documented and justified to ensure transparency.

#### Step 8: nutrient gap classification

Based on these assessments, we classified nutrient gaps into the following 2 categories: clear nutrient gaps, where both burden and evidence certainty ratings were at least *moderate*, and potential nutrient gaps, where burden was *high* or *moderate,* but evidence certainty was low.

### Identification of best food sources

To identify the best food sources to address the identified nutrient gaps, we used anonymized secondary data from the 2019‒2020 Malawi IHS5 [17]. A detailed description of the survey is available from https://microdata.fao.org/index.php/catalog/1760/pdf-documentation. The dataset has a sample size of 11,434 households, from which we selected a subset of 9296 households with ≥1 WRA for this analysis. Food consumption in IHS5 was collected as a recall from a predefined list of 135 food items over a period of 7 d. We matched these data to available food composition data for micronutrient profiling, using the food composition table (FCT) for Malawi as the primary source [18] and complementing it with additional FCTs, such as the Kenyan and Western Africa FCTs, where necessary to fill gaps in nutrient information [19,20]. Using these sources, we estimated apparent intake, defined as the approximated amount of a food (and its nutrients) that a consumer may ingest, calculated through indirect means such as Household Consumption and Expenditure Survey (HCES) or through secondary analysis of reports of food availability, access, and acquisition [21]. This approach provided a practical means of identifying food sources with the highest potential to contribute meaningfully to closing observed nutrient gaps among Malawian WRA.

Apparent food intake was estimated as the quantity of each food item reportedly consumed by the household, expressed in g/d, and then adjusted using the adult female equivalent (AFE) metric. The AFE metric assumes that apparent food intake is distributed in proportion to the energy requirements of each household member, standardized to the nonpregnant, non-lactating 18- to 29-y-old female who serves as a reference family member or 1 AFE [22]. This adjustment allowed us to approximate the share of household food available to WRA and to evaluate whether the consumed quantities were sufficient to meet dietary needs. The median nutrient intake per day per AFE was also analyzed to verify whether the identified food sources were effectively contributing to the daily micronutrient intake of WRA.

The apparent intake was represented by the following Equation 1:

(1) $$\text{Apparent}\;\text{intake}\;\left(\frac{\frac{g}{d}}{\text{AFE}}\right)=\left(\frac{\text{Quantity}\;\text{of}\;\text{food}\;\text{consumed}\;\text{by}\;\text{the}\;\text{households}\;\left(\frac{g}{d}\right)\;}{\;\text{AFE}}\right)$$

The estimated apparent intakes were compared against the harmonized average requirement (H-AR) values for an adult female, nonpregnant, non-lactating, aged 18 to 29 y to determine the presence and magnitude of nutrient gaps for Ca, Fe, Se, and Zn. The H-AR is defined as the mean daily nutrient intake that is estimated to meet the requirements of half of the healthy individuals in a particular life stage and gender group [23]. The H-ARs for Ca, Fe, Se, and Zn were 860 mg, 22.4 mg, 45 *μ*g, and 10.2 mg, respectively [23]. The H-AR values for Fe and Zn incorporate assumptions about fractional absorption, with Fe estimated at 5% absorption and Zn based on low bioavailability typical of unrefined, cereal-based diets, which are consistent with prevailing dietary patterns in Malawi.

Foods included in the analysis met the following 2 key criteria: first, the nutrient content of the food had the potential to provide ≥50% of the nutrient needs of a WRA as determined using the Malawi FCT (per 100 g edible portion), expressed by the following Equation 2:

(2) $$\text{Nutrient}\;\text{requirement}\;\text{met}\;\left(\geq 50\%\right)=\left(\frac{\text{Nutrient}\;\text{content}\;\text{of}\;\text{food}\;}{\;H-\text{AR}\;\text{of}\;\text{WRA}\;\text{for}\;\text{specific}\;\text{nutrient}}\right)\times 100$$

Foods meeting or exceeding 50% of the H-AR were considered strong candidates for addressing identified nutrient gaps. Second, there was adequate data on local food consumption to make reliable estimates. Foods that satisfied both criteria were identified as the best food sources for addressing the nutrient gaps. The threshold of ≥50% of the H-AR per 100 g edible portion was selected to identify foods with high nutrient density and strong potential to meaningfully contribute to closing nutrient gaps. Although a 100 g reference portion may not reflect typical consumption amounts for all foods, it provides a standardized basis for comparing nutrient density across diverse food items. Sensitivity analyses using lower thresholds (i.e., 30%‒40%) were initially explored; however, the ≥50% threshold was retained to focus on foods with the greatest potential impact, given the magnitude of the observed nutrient gaps and generally low consumption levels. These findings should therefore be interpreted as identifying candidate foods with high potential.

We further examined the extent to which the identified key dietary sources were already being consumed by assessing their coverage and apparent consumption quantities. Coverage was estimated as the percentage of households consuming any quantity of the selected food items. This analysis was required to determine whether nutrient-rich foods were extensively consumed or if their consumption was limited to a small subset of the population. All data cleaning, processing, transformation, and analysis were performed in RStudio (version 4.1.3, R Foundation for Statistical Computing), and the codebook can be shared on request.

## Results

A total of 67 qualifying data points from 14 evidence sources were identified during the literature search, 58 of which qualified for the quantitative burden score (Supplemental Table 3). The evidence sources were from the years 2015 to 2024. There were variations in the available data points across micronutrients and evidence types, with some nutrients supported by multiple indicators from several evidence types, whereas others had sparse or uneven coverage.

### Nutrient gaps

Table 1 shows the nutrient (mineral and vitamin) gap burden and certainty ratings for all 17 assessed micronutrients. Based on the final combined ratings, clear nutrient gaps were identified for Ca, Fe, Se, and Zn, but not for any of the vitamins. All the mentioned micronutrients had a burden rating of “high” and a certainty rating of “moderate.” An exception was Ca, which had a final burden of “high” and a certainty rating of “high.” The assessment also identified potential nutrient gaps for P and vitamin B1, vitamin B12, and vitamin C, but more data are needed to confirm their public health burden.

**TABLE 1:**
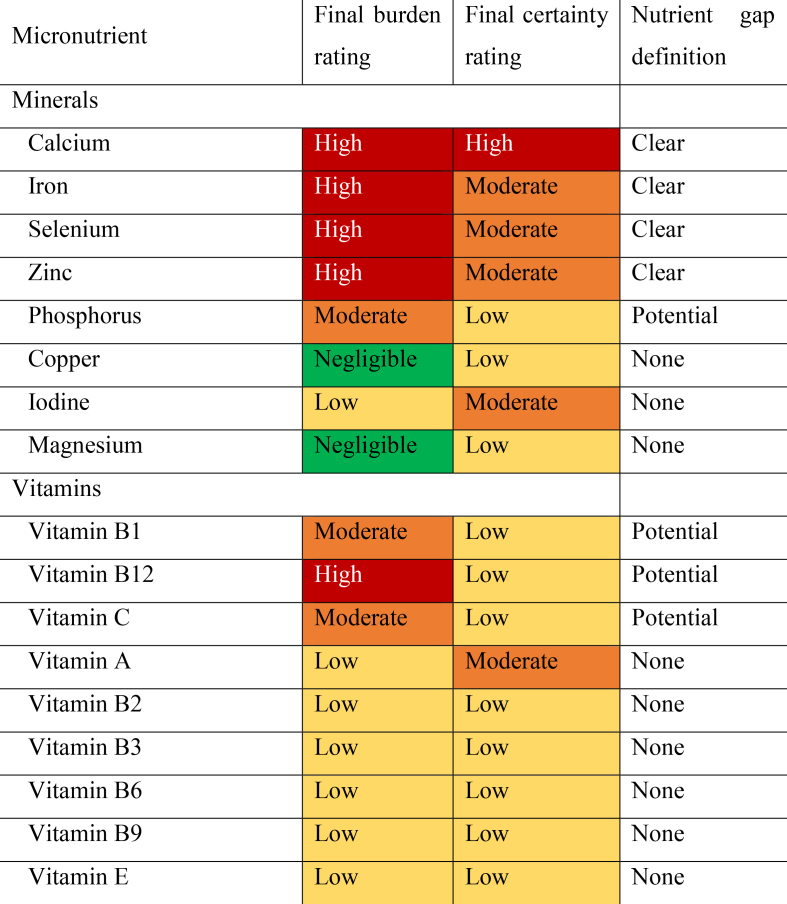
Final ratings and gap definition for assessed micronutrients

**Note**: Nutrient gap definitions follow the CONGA framework (13). “Clear” denotes strong evidence of a nutrient gap; “Potential” indicates suggestive but uncertain evidence; and “None” indicates that no meaningful nutrient gap was identified.

After qualitative validation with experts and stakeholders, adjustments were made to the quantitative burden ratings for 6 of the 17 assessed micronutrients. Except for Zn, these adjustments resulted in a reduction in the severity of the burden ratings. For example, the initial quantitative rating for vitamin A was revised from “moderate” to “low” after considering existing interventions such as fortification and biofortification efforts. Similarly, vitamin B3 and vitamin B9 were reclassified from “moderate” to “low,” supported by evidence from the micronutrient action policy support tool (https://maps.micronutrient.support) indicating that requirements are largely met at the population level. Vitamin B2 and vitamin E were downgraded from “high” to “low” due to limited available evidence.

In contrast, certainty ratings generally improved after qualitative assessment. Certainty increased from “low” to “moderate” for Fe, vitamin A, Zn, and I, reflecting more consistent evidence across data sources. Ca was the only micronutrient for which certainty increased from “low” to “high,” as the available evidence consistently indicated a high burden.

### Evidence gaps

Across the 5 CONGA evidence types, notable gaps were observed in the availability and distribution of data points (Supplemental Table 3). The nutrient adequacy of household diets provided the largest share of evidence, contributing 26 data points, of which 22 qualified for the quantitative burden score. In contrast, nutrient adequacy of individual diets had the least number of data points (*n* = 4), although all qualified for the quantitative burden score. A total of 10 data points were identified for the biological, clinical, and functional evidence type, most of which were for Fe (*n* = 3) and Zn (*n* = 2). Notably, no qualifying biological and functional evidence was available for Ca, P, Cu, Mg, and vitamin B1, vitamin B2, vitamin B3, vitamin B6, vitamin C, and vitamin E. The nutrient adequacy of national food supplies evidence type contributed 14 data points, covering all nutrients except I, Se, and vitamin E. Nutrient-informative food-group intake of individuals or households had 9 data points, most of which were for vitamin A, Ca, and I.

### Best food sources

We identified small sun-dried fish, medium sun-dried fish, small smoked fish, medium smoked fish, powdered milk, pork, goat, egg, and gathered wild green leaves as the best sources capable of providing ≥50% of the dietary requirements of WRA (per 100 g edible portion) for ≥1 of the identified nutrient gaps (Table 2). Se had the widest range of food sources—8 of 9 identified foods met at least half of the H-AR for this nutrient, followed by Ca, for which 6 of 9 foods were adequate sources. In contrast, fewer foods could meaningfully contribute to addressing the Zn gap. Among all the foods evaluated, small sun-dried fish had the greatest potential to meet at least half of the WRA’s requirements for all 4 nutrient gaps (Ca, Fe, Se, and Zn).

**TABLE 2:**
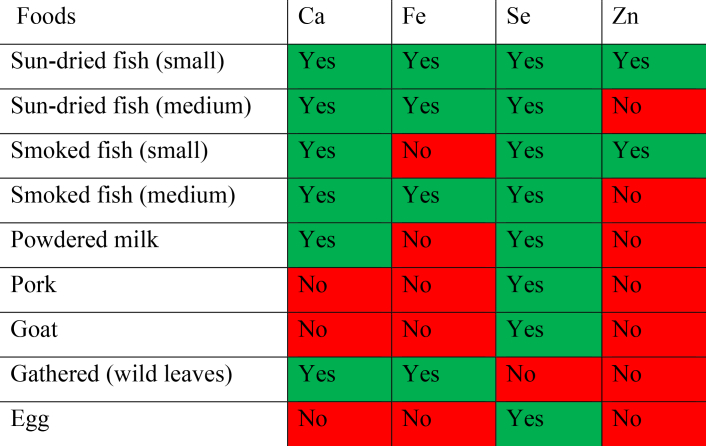
Selected foods with the potential to meet ≥50% of the nutrient needs of women of reproductive age for ≥1 priority nutrient

**Note:** Foods were included if their nutrient content per 100 g edible portion provided ≥50% of the harmonized average requirement (H-AR) for at least one priority nutrient. "Yes" indicates that the food provides ≥50% of the harmonized average requirement (H-AR) per 100 g edible portion for the specified nutrient, whereas "No" indicates that it does not.

The coverage and consumption quantities of the identified key dietary sources are presented in Table 3. Generally, coverage was low, with no single food apparently consumed by more than half of households in the past week. Eggs had the highest coverage (42%), followed by small sun-dried fish (36%). Despite their relatively higher coverage, food consumption quantities were frequently insufficient to meet at least half of the daily nutrient gaps for WRA. For example, although having the highest coverage, eggs were consumed in small quantities (14 g/d per AFE), implying that their contribution to closing the nutrient gaps would be minimal despite being a viable food source. Our analysis further revealed distinct patterns in animal-source food consumption by residence and socioeconomic position (SEP) and seasonal distribution (Supplemental Figure 1 and Supplemental Table 4).

**TABLE 3:** Coverage (percentage) and median consumption (grams/day per adult female equivalent) of key dietary sources over the previous 7 d

Food item	Coverage (%)	Median consumption (g/d per AFE)
Egg	42.2	13.7
Sun-dried fish (small)	35.8	7.5
Gathered wild green leaves	22.5	17.7
Goat	13.9	24.8
Sun-dried fish (medium)	12.1	6.6
Smoked fish (small)	11.0	6.6
Smoked fish (medium)	10.6	7.6
Powdered milk	9.2	8.1
Pork	7.9	22.9

Abbreviation: AFE, adult female equivalent.

Reproduced from data source: Malawi IHS5 [17] with permission.

### Apparent intake of Ca, Fe, Se, and Zn based on Malawi’s IHS5

**FIGURE 1:**
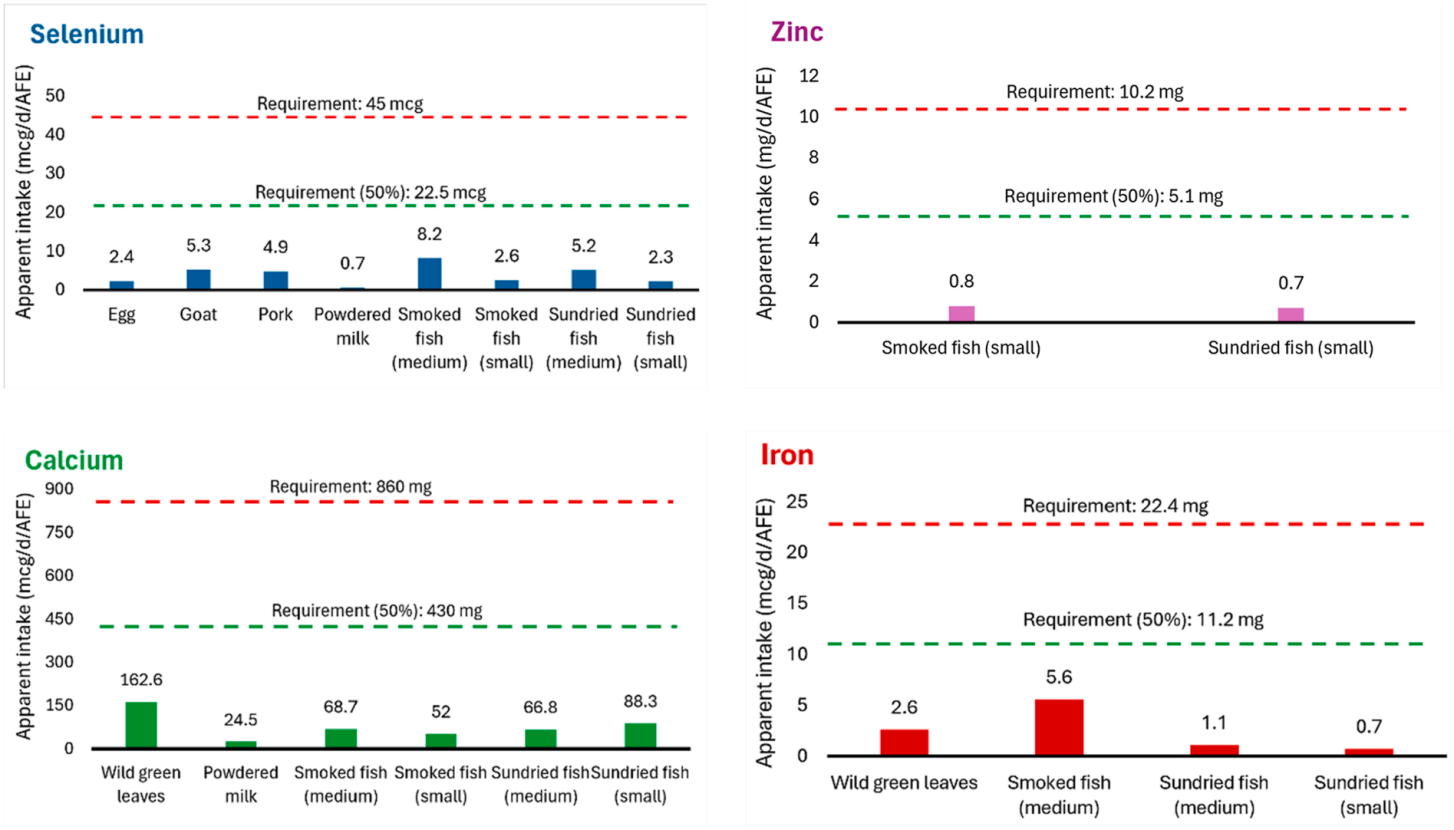
Apparent intake of Se, Zn, Fe, and Ca from identified best food sources. Different colored bars represent individual food sources contributing to apparent micronutrient intake, the green dashed line indicates 50% of the harmonized average requirement (H-AR) WRA), whereas the red dashed line indicates 100% of the H-AR, WRA requirement sourced from Allen et al. [23], 2019. Reproduced from data sources: Malawi IHS5 [17] with permission. Ca, calcium; Fe, iron; IHS5, Fifth Integrated Household Survey; Se, selenium; Zn, zinc.

Figure 1 illustrates the apparent intake of Ca, Fe, Se, and Zn by best food sources. None of the foods, at their current consumption levels, provided ≥50% of the WRA daily requirements for any of the 4 nutrients. Medium smoked fish contributed comparatively higher amounts of Fe and Se, whereas gathered wild green leaves were leading sources of Ca. The apparent intake of Zn was particularly limited. In a hypothetical scenario where individuals (in terms of AFE) consumed all identified nutrient-rich foods simultaneously, apparent intake would meet only 31.6 *μ*g of Se (70% of requirement) and 462.9 mg of Ca (54% of requirement) and fall even lower for Fe (10 mg; 45% of requirement) and Zn (1.5 mg; 15% of requirement). Even under this unrealistic best-case scenario, none of the nutrients achieved 100% of daily requirements. This scenario emphasizes that current consumption levels are insufficient to close the identified nutrient gaps, particularly Fe and Zn.

### Food group contribution to key micronutrient intakes

**FIGURE 2:**
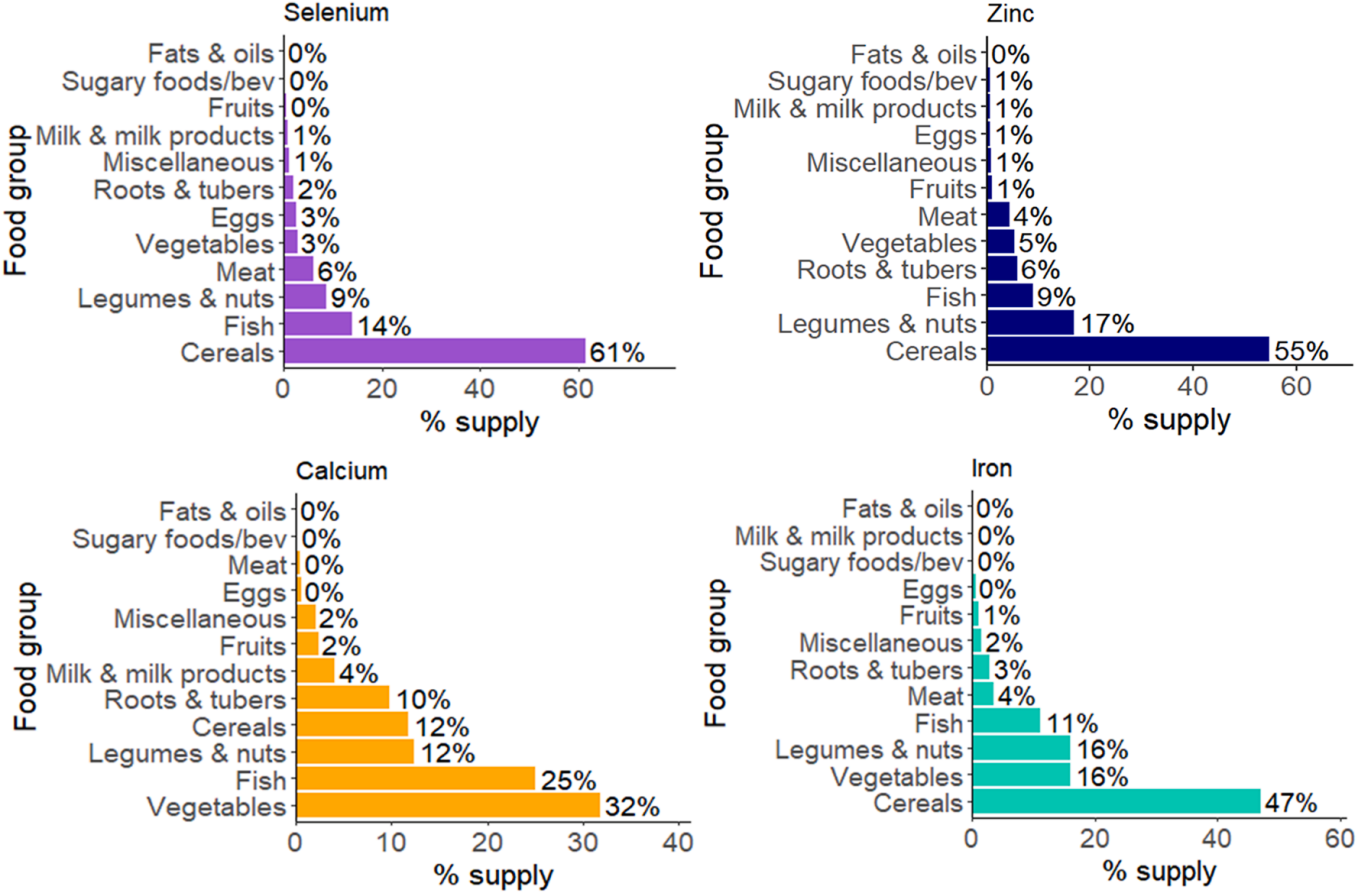
Relative contribution of various foods to apparent intake of Se, Zn, Fe, and Ca. Different colors represent the food groups contributing to micronutrient intake (e.g., cereals, vegetables, fish, legumes and nuts, roots and tubers, fruits, milk and milk products, meat, and other food groups). Reproduced from data sources: Malawi IHS5 [17] with permission. Ca, calcium; Fe, iron; IHS5, Fifth Integrated Household Survey; Se, selenium; Zn, zinc.

In addition to identifying specific food sources, we performed food group analysis to understand how different food groups contribute to apparent intake of Ca, Fe, Se, and Zn (Figure 2). Results show that except for Ca, whose main source was vegetables, the main sources of Se (61%), Zn (55%), and Fe (47%) were cereals. Fish were the fourth, second, second, and third best source of Fe, Ca, Se, and Zn, respectively. However, the difference between the plant-based source and fish was 7 percentage points for Ca, 36 percentage points for Fe, 45 percentage points for Se, and 46 percentage points for Zn. This means that regarding animal-source foods (ASFs), fish were the most accessible source, but intake was substantially low.

## Discussion

This study aimed to assess the public health significance of MNDs among WRA in Malawi using existing evidence by applying the CONGA methodology and to propose food-based sources to address identified nutrient gaps, leveraging food consumption data from Malawi’s IHS5. Our assessment revealed clear nutrient gaps for Ca, Fe, Se, and Zn, for which both the burden and certainty were rated at least moderate. Ca stood out with high burden and high certainty, indicating a pervasive deficiency. These nutrient gaps carry important health implications for WRA: Ca deficiency heightens risk of hypertensive disorders during pregnancy and contributes to poor long-term bone health problems [24]; inadequate Fe and Zn weaken immune function, reduce physical work capacity, and are associated with adverse pregnancy outcomes [25,26]; and Se deficiency contributes to oxidative stress and impaired thyroid function [27]. In contrast, P, vitamin B1, vitamin C, and vitamin B12 have potential for concern, but more data are needed, particularly for the first 3 micronutrients, to validate the significance of these burdens.

The best dietary sources of bioavailable Fe, Zn, Ca, and Se are ASFs [28]. However, in Malawi, where diets are primarily plant-based, the main contributors of these nutrients are cereals [29], which are laden with the mineral-chelating effects of phytate [30]. Elsewhere, mineral deficiencies due to habitual consumption of plant-based diets, which have poor bioavailability, have been documented for Fe [31], Zn [32], Cu [33], and vitamin B12 [34]. The low intake of ASFs observed in the Malawian diet provides a plausible explanation for the clear nutrient gaps identified in this study (i.e. Fe, Zn, Ca, and Se), reinforcing the need to strengthen access to and consumption of nutrient-dense ASFs.

We observed that eggs and small- and medium-sized sun-dried fish were the most frequently consumed ASFs; however, consumption was highly skewed toward wealthier and urban households (Supplemental Table 4). This is attributed to the high cost of these foods, which are beyond the reach of many Malawian households [35], the majority (59%) of whom live in multidimensional poverty [36]. According to Bwanaisa and Hendriks [35], beans, despite being rich in phytate, were the cheapest source of protein for Malawians between 2017 and 2021. This means that poverty is a key factor driving MNDs, particularly minerals, which calls for practical efforts to improve household incomes coupled with nutrition education to improve access, affordability, and consumption of ASFs. One way could be through the promotion of small livestock interventions targeted at low SEP and rural households [36,37].

Several foods were identified as the best food sources for addressing the nutrients with clear gaps (Ca, Fe, Se, and Zn). However, despite their nutrient density, current intakes and household coverage of these foods remain very low. Our analysis showed that even when combining several of the identified foods, individuals (in terms of the AFE) would only meet ∼50% of the daily requirements for Se and Ca, but far less for Zn and Fe. However, even such an impractical scenario would not meet the requirement of any of these nutrients because of the low intakes.

### Policy implications and future directions

Our study findings highlight that addressing micronutrient gaps among Malawian WRA will require actions that extend beyond dietary diversity alone. Although several locally available foods have the potential to contribute to improved intake of Ca, Fe, Se, and Zn, current consumption levels are too low to meaningfully reduce deficiencies. This points to the need for integrated, system-level strategies that improve both the availability, accessibility, and affordability of nutrient-dense foods.

The use of HCES data in this study provides a practical entry point for translating evidence into action by identifying nutrient-dense foods that are locally available yet underconsumed. However, the value of this approach lies in how these insights are operationalized. Moving from identification to implementation requires several next steps. These include assessing the affordability, availability, and seasonal stability of the identified foods, particularly among vulnerable populations; evaluating the feasibility of increasing their supply through strengthened value chains; and identifying demand-side barriers such as cultural preferences and intra-household food allocation that may limit consumption among WRA. Integrating these foods into existing programs such as social protection, school feeding, and community-based nutrition interventions could further support increased intake.

From a policy perspective, and building on these implementation considerations, Malawi’s success with vitamin A fortification offers a useful model for exploring fortification or biofortification options for other key nutrients, particularly Fe and Zn. It is important to note that Malawi already has mandatory large-scale food fortification (LSFF) policies, including fortification of sugar and cooking oil with vitamin A and wheat flour with a premix containing Fe, Zn, and several B vitamins. These existing interventions were considered during the qualitative assessment stage of the CONGA methodology, which contributed to the downgrading of the vitamin A burden from moderate to low. However, despite mandatory fortification of wheat flour with Fe and Zn, clear nutrient gaps for these minerals persist. This suggests potential challenges related to coverage and compliance and highlights the need to strengthen monitoring and enforcement of existing fortification programs. Additionally, expanding LSFF to include other widely consumed food vehicles may further enhance its effectiveness.

Furthermore, strengthening value chains for small fish, one of the most nutrient-dense foods identified in this study, could help increase their accessibility in rural and low-income markets. Complementary interventions such as small livestock promotion (e.g., chickens and rabbits), social protection measures that enhance purchasing power, and targeted nutrition education could also help households consume more ASFs.

Future research should prioritize nationally representative biomarker surveys for nutrients such as vitamin B2 and Se. Studies exploring the affordability and seasonal availability of identified key nutrient-dense foods would further strengthen the evidence base needed for effective policy and programming.

### Study strengths and limitations

This study has several notable strengths. First, it is one of the few efforts in Malawi to apply the CONGA methodology, allowing for a structured synthesis of fragmented evidence across multiple indicators and data sources. Second, the use of nationally representative household consumption data from the IHS5 enabled the identification of realistic, context-specific food sources with the potential to address identified nutrient gaps. Third, involving national technical working groups in reviewing and validating the evidence enhanced contextual relevance.

However, the study also has limitations. First, our use of the AFE metric, because of the absence of individual-level dietary data, may be inaccurate because it assumes standardized intra-household food distribution based solely on age and sex, which would not capture real-world disparities in actual consumption patterns, cultural biases, or individual nutritional needs in Malawi. Second, the paucity of nationally representative biomarker data for other micronutrients impedes definitive assessment of deficiency prevalence, which means that the observed gaps may reflect evidence limitations rather than true absence of deficiencies. These limitations strengthen the need for future research pairing individual dietary surveys with biomarker analysis to accurately characterize Malawi’s micronutrient challenges among WRA.

In conclusion, our analysis has revealed critical evidence gaps, with few studies employing biomarkers or individual dietary assessments to evaluate micronutrient status. Nevertheless, we identified clear deficiencies in Ca, Fe, Se, and Zn among Malawian WRA. Although Fe, Zn, and Se deficiencies were consistent with existing literature, the Ca gap represents, to our knowledge, a novel finding of public health concern. Even when considering locally available foods capable of meeting ≥50% of WRA nutrient requirements, consumption patterns vary. These findings underscore the following 3 programmatic priorities: *1*) targeted nutrition education promoting dietary diversification, *2*) improved access to nutrient-rich foods through market-based and agricultural interventions, and *3*) scaling evidence-based fortification and biofortification programs for nutrients with established deficiencies. These interventions should be layered on strong poverty reduction strategies that would increase disposable income for improved affordability of better dietary sources (e.g., ASFs) of Se, Ca, Zn, and Fe. Additional research is warranted to investigate potential gaps in other micronutrients where current evidence remains inconclusive and extend to other demographic groups vulnerable to MNDs.

## Author contributions

The authors’ responsibilities were as follows – ZK-C: conceptualized and designed the study; ZK-C, BM-M, GO: curated the evidence base, populated the datasets, and conducted the initial analyses; AAK: reviewed and validated the preliminary nutrient gap classifications; ZK-C, BM-M, GO, AAK: contributed to reassessing the certainty of evidence for each nutrient gap and collectively conducted the qualitative validation; ZK-C, AAK: drafted the initial manuscript; and all authors: read and approved the final manuscript.

## Data availability

This study used summary prevalence data from previously published micronutrient studies available in the public domain and anonymized secondary data from the 2019‒2020 Malawi Fifth Integrated Household Survey, which are publicly available through the World Bank Microdata Library at https://microdata.worldbank.org/index.php/catalog/3818. The Excel workbook containing all Comprehensive Nutrient Gap Assessment scoring procedures and nutrient gap calculations is provided as Supplemental Data 1 (Excel workbook).

## Declaration of Generative AI and AI-assisted technologies in the writing process

The authors declare that no generative AI or AI-assisted technologies were used in the writing of this manuscript.

## Funding

This study was funded through Micronutrient Forum’s Data Innovation Alliance small grants program. The funder did not have a role in the conceptualization, design, data curation, analysis, decision to publish, or preparation of the manuscript.

## Conflict of interest

The authors report no conflicts of interest.
