# Supplementary material for: From Evidence to Action: Addressing Micronutrient Gaps among Women of Reproductive Age in Malawi Using the Comprehensive Nutrient Gap Assessment Framework
Source: Curr Dev Nutr. 2026 Jul 11;10(8):109436. doi: 10.1016/j.cdnut.2026.109436 (PMC13476496; doi:10.1016/j.cdnut.2026.109436)
Supplement: Supplementary file 1 [file mmc1.docx]

**Supplemental Materials**

**Table of Contents**

[**Supplemental Table 1:** Prevalence ranges for implied nutrient gap burden scores ^*^	2](#_Toc236683104)

[**Supplemental Table 2:** Certainty-of-evidence rating criteria ^*^	3](#_Toc236683105)

[**Supplemental Table 3:** Number of data points by evidence type and nutrient (number qualified for the quantitative burden score)	4](#_Toc236683106)

[**Supplemental Table 4:** Coverage (%) of egg and fish consumption across residence and socioeconomic position in Malawi over the previous 7 days	5](#_Toc236683107)

[**Supplemental Figure 1:** Seasonal distribution of apparent intake (g/day per AFE) of selected food items by residence	6](#_Toc236683108)

# **Supplemental Table 1:** Prevalence ranges for implied nutrient gap burden scores ^*^

| **Indicator** | **Age** | **Implied nutrient burden rating** | | | |
| --- | --- | --- | --- | --- | --- |
|  |  | **Negligible** | **Low** | **Moderate** | **High** |
| **Biomarkers** |  |  |  |  |  |
| Iron |  |  |  |  |  |
| Serum ferritin (< 12 mg/l) | < 5 y | < 3% | 3–9% | 10–19% | ≥ 20% |
| Vitamin A |  |  |  |  |  |
| Serum retinol (< 0.7 mmol/l) | 6–71 m | < 3% | 3–9% | 10–19% | ≥ 20% |
| Zinc |  |  |  |  |  |
| Serum zinc (< 9.9 mmol/l) | < 10 y | < 3% | 3–9% | 10–19% | ≥ 20% |
| Folate |  |  |  |  |  |
| Serum folate (< 10 nmol/l) | All | < 3% | 3–9% | 10–19% | ≥ 20% |
| Vitamin B12 |  |  |  |  |  |
| Plasma B12 (< 150 pmol/l) | All | < 3% | 3–9% | 10–19% | ≥ 20% |
| Vitamin D |  |  |  |  |  |
| Serum 25(OH)D <50 nmol/L | All | < 3% | 3–9% | 10–19% | ≥ 20% |
| Iodine |  |  |  |  |  |
| Urinary iodine concentration (UIC) (< 100 mg/l) | ≥ 6 y | < 25% | 25–49% | 50–74% | ≥ 75% |
| Median UIC (mg/l) | ≥ 6 y | ≥ 150 | 100–150 | 50–99 | < 50 |
| Total goiter rate | 6–12 y | < 3% | 3–9% | 10–19% | ≥ 20% |
| **Inadequate intake or availability** |  |  |  |  |  |
| Iron, vitamin A, zinc, folate, vitamin B12 | All | < 5% | 5–14% | 15–24% | ≥ 25% |
| Calcium, niacin, thiamine, vitamin B6 | All | < 5% | 5–19% | 20–49% | ≥ 50% |
| Vitamin C | All | < 10% | 10–29% | 30–49% | ≥ 50% |
| **Nutrient-informative food group intake** |  |  |  |  |  |
| Vitamin A-rich foods (past 24 hours) | 6–23 m | >90% | 75-90% | 60-74% | <60% |
| Iron-rich foods (past 24 hours) | 6–23 m | > 70% | 60–70% | 50–59% | < 50% |
| Household iodized salt coverage | All | > 90% | 75–90% | 60–74% | < 60% |
| Household adequately iodized salt coverage (≥ 15 ppm) | All | > 80% | 65–80% | 50–64% | < 50% |

*Abbreviation*: UIC, urinary iodine concentration; m, months; y, years.

^*^Adapted from Beal T, White JM, Morris SS, Arsenault JE, Okronipa H, Hinnouho G marino. Comprehensive nutrient gap assessment (CONGA): A method for identifying the public health significance of nutrient gaps. Nutr Rev. 2021;79:4–15. Available from: https://doi.org/10.1093/nutrit/nuaa140

# **Supplemental Table 2:** Certainty-of-evidence rating criteria ^*^

| **Low** | **Moderate** | **High** |
| --- | --- | --- |
| ≥1 qualifying data point meeting minimum inclusion criteria for the quantitative nutrient gap burden calculation b | - ≥1 data point with a weight score ^c^ of 51–80 and no disagreements ^d^ with any data point with a weight score > 25 - Or ≥2 data points with a weight score 25–50 and no disagreements with any data point with a weight score > 25 - Or ≥3 data points meeting minimum criteria thresholds, one of which whose weight score is ≥ 15, and no disagreements with any rating | - ≥1 data point with a weight score > 80 and no disagreements with any data point with a weight score > 50 - ≥2 data points with a weight score > 50 and no disagreements with any data point with a weight score > 50 |

* Adapted from Beal T, White JM, Morris SS, Arsenault JE, Okronipa H, Hinnouho G marino. Comprehensive nutrient gap assessment (CONGA): A method for identifying the public health significance of nutrient gaps. Nutr Rev. 2021;79:4–15. Available from: https://doi.org/10.1093/nutrit/nuaa140

# **Supplemental Table 3:** Number of data points by evidence type and nutrient (number qualified for the quantitative burden score)

|  | **Evidence type** | | | | | |  |
| --- | --- | --- | --- | --- | --- | --- | --- |
| **Micronutrients assessed** | **Biological, clinical and functional markers** | **Nutrient adequacy of individual diets** | **Nutrient adequacy of household diets** | **Nutrient adequacy of national food supplies** | **Nutrient-informative food-group intake of individuals or households** | **Other** | **Total** |
| Calcium | 0(0) | 0(0) | 2(1) | 1(1) | 2(2) | 0(0) | 5(4) |
| Copper | 0(0) | 0(0) | 1(1) | 1(1) | 0(0) | 0(0) | 2(2) |
| Iodine | 1(1) | 0(0) | 0(0) | 0(0) | 2(2) | 0(0) | 3(3) |
| Iron | 2(1) | 1(1) | 3(2) | 1(1) | 1(1) | 3(0) | 11(6) |
| Magnesium | 0(0) | 0(0) | 1(1) | 1(1) | 0(0) | 0(0) | 2(2) |
| Phosphorus | 0(0) | 0(0) | 1(1) | 1(1) | 0(0) | 0(0) | 2(2) |
| Selenium | 1(1) | 0(0) | 2(1) | 0(0) | 0(0) | 0(0) | 3(2) |
| Zinc | 3(3) | 1(1) | 3(2) | 1(1) | 0(0) | 0(0) | 8(7) |
| Vitamin A | 1(1) | 1(1) | 3(3) | 1(1) | 3(3) | 1(0) | 10(9) |
| Vitamin B1 | 0(0) | 0(0) | 1(1) | 1(1) | 0(0) | 0(0) | 2(2) |
| Vitamin B2 | 0(0) | 0(0) | 1(1) | 1(1) | 0(0) | 0(0) | 2(2) |
| Vitamin B3 | 0(0) | 0(0) | 1(1) | 1(1) | 0(0) | 0(0) | 2(2) |
| Vitamin B6 | 0(0) | 0(0) | 1(1) | 1(1) | 0(0) | 0(0) | 2(2) |
| Vitamin B9 | 1(1) | 1(1) | 3(3) | 1(1) | 0(0) | 0(0) | 6(6) |
| Vitamin B12 | 1(1) | 0(0) | 1(1) | 1(1) | 1(1) | 0(0) | 4(4) |
| Vitamin C | 0(0) | 0(0) | 1(1) | 1(1) | 0(0) | 0(0) | 2(2) |
| Vitamin E | 0(0) | 0(0) | 1(1) | 0(0) | 0(0) | 0(0) | 1(1) |
| **Total** | **10(9)** | **4(4)** | **26(22)** | **14(14)** | **9(9)** | **4(0)** | **67(58)** |

*Quantitative burden score:* A weighted mean used to estimate the public health significance of a nutrient gap, calculated as the sum of the implied nutrient gap scores multiplied by their respective metadata weights, divided by the sum of the weights.

# **Supplemental Table 4:** Coverage (%) of egg and fish consumption across residence and socioeconomic position in Malawi over the previous 7 days

| **Population** | **Households** | **Egg** | **Sun-dried fish small** | **Sun-dried fish medium** |
| --- | --- | --- | --- | --- |
|  | **n** | **%** | | |
| Residence and socioeconomic position (SEP) by quintile of total annual household expenditure per capita | | | | |
| **Rural** | **7528** | **36** | **35** | **10** |
| Lowest SEP | 1668 | 12 | 30 | 4 |
| Lower Middle SEP | 1637 | 24 | 34 | 9 |
| Middle SEP | 1558 | 35 | 36 | 10 |
| Higher Middle SEP | 1448 | 49 | 37 | 14 |
| Highest SEP | 1217 | 71 | 39 | 17 |
| **Urban** | **1768** | **67** | **39** | **20** |
| Lowest SEP | 401 | 36 | 44 | 12 |
| Lower Middle SEP | 391 | 62 | 42 | 21 |
| Middle SEP | 373 | 75 | 40 | 25 |
| Higher Middle SEP | 333 | 85 | 33 | 26 |
| Highest SEP | 270 | 90 | 32 | 17 |

**Data source:** Malawi IHS5

# **Supplemental Figure 1:** Seasonal distribution of apparent intake (g/day per AFE) of selected food items by residence

**Data source:** Malawi IHS5
